# Characteristics of ascitic fluid from patients with suspected spontaneous bacterial peritonitis in emergency units at a tertiary hospital

**DOI:** 10.1590/S1516-31802011000500006

**Published:** 2011-09-01

**Authors:** Thiago José Buer Reginato, Marcelo José Andrade Oliveira, Luiz César Moreira, Antonieta Lamanna, Milena Marques Pagliarelli Acencio, Leila Antonangelo

**Affiliations:** I Medical Student. Faculdade de Medicina da Universidade de São Paulo (FMUSP), São Paulo, Brazil.; II MD. Clinical Pathologist, Clinical Laboratory, Department of Pathology, LIM 03, Hospital das Clínicas (HC), Faculdade de Medicina da Universidade de São Paulo (FMUSP), São Paulo, Brazil.; III BSc. Biologist. Clinical Laboratory, Department of Pathology, LIM 03, Hospital das Clínicas (HC), Faculdade de Medicina da Universidade de São Paulo (FMUSP), São Paulo, Brazil.; IV BSc, PhD. Biologist, Heart Institute, Faculdade de Medicina da Universidade de São Paulo (FMUSP), São Paulo, Brazil.; V MD, PhD. Clinical Pathologist and Professor, Clinical Laboratory, Department of Pathology, LIM 03, Hospital das Clínicas (HC), Faculdade de Medicina da Universidade de São Paulo (FMUSP), São Paulo, Brazil.

**Keywords:** Ascitic fluid, Infection, Paracentesis, Cytology [subheading], Peritonitis, Líquido ascítico, Infecção, Paracentese, Citologia, Peritonite

## Abstract

**CONTEXT AND OBJECTIVE:**

Spontaneous bacterial peritonitis (SBP) is a complication of ascites, especially in cirrhosis. Ascitic fluid with 250 or more neutrophils/mm^3^ is an acceptable criterion for diagnosis, even when bacterial fluid cultures are negative. The aims here were to estimate SBP frequency among emergency room patients based on cellular criteria and evaluate the biochemical profile of these fluids.

**DESIGN AND SETTING:**

Retrospective study at a public tertiary hospital.

**METHODS:**

Laboratory records of patients with ascites attended in emergency rooms between November 2001 and November 2006, from whom ascitic fluid samples were sent to the laboratory due to suspected SBP, were evaluated. The 691 samples included were divided into group A (presumed SBP: ≥ 250 neutrophils/mm^3^; n = 219; 31.7%) and group B (no presumed SBP: < 250 neutrophils/mm^3;^ n = 472; 68.3%). Patients’ sex and age; ascitic fluid characteristics (numbers of neutrophils, leukocytes and nucleated cells); bacteriological characteristics; and protein, lactate dehydrogenase, adenosine deaminase and glucose concentrations were evaluated.

**RESULTS:**

Among group A cultured samples, 63 (33.8%) had positive bacterial cultures with growth of pathogens commonly associated with SBP. In total, the group A samples showed higher lactate dehydrogenase levels than seen in the group B samples. The latter presented predominance of lymphocytes and macrophages.

**CONCLUSION:**

Among the ascitic fluid samples with clinically suspected SBP, 31.7% fulfilled the cellular diagnostic criteria. Positive bacterial isolation was found in 33.8% of the cultured samples from the presumed SBP group.

## RESUMO

**CONTEXTO E OBJETIVO:**

Peritonite bacteriana espontânea (PBE) é uma complicação da ascite, especialmente na cirrose. Líquido ascítico com 250 ou mais neutrófilos/mm^3^ é um critério aceitável para o diagnóstico, mesmo com cultura bacteriana negativa. Os objetivos foram estimar a frequência de PBE em pacientes atendidos na sala de emergência, baseando-se no critério celular e avaliar o perfil bioquímico desses líquidos peritoneais.

**TIPO DE ESTUDO E LOCAL:**

Estudo retrospectivo em hospital público terciário.

**MÉTODOS:**

Foram avaliados registros laboratoriais de pacientes com ascite atendidos no setor de emergência entre novembro de 2001 e novembro de 2006, cujas amostras de líquido ascítico foram encaminhadas ao laboratório por suspeita de PBE. As 691 amostras incluídas foram divididas em grupo A (PBE presumida: ≥ 250 neutrófilos/mm^3^; n = 219; 31.7%) e grupo B (Ausência de PBE presumida: < 250 neutrófilos/mm^3;^ n = 472; 68.3%). Também foram avaliados sexo e idade dos pacientes além de características dos líquidos ascíticos: número de neutrófilos, leucócitos e células nucleadas; bacteriologia; e concentrações de proteínas, desidrogenase láctica, adenosina deaminase e glicose.

**RESULTADOS:**

Das amostras cultivadas do grupo A, 63 (33,8%) tiveram cultura bacteriana positiva com crescimento de patógenos comumente associados à PBE. O total de amostras do grupo A exibiu maiores níveis de desidrogenase lática que as do grupo B. Este último demonstrou predomínio de linfócitos e macrófagos.

**CONCLUSÃO:**

Dos líquidos ascíticos com suspeita clínica de PBE, 31.7% preencheram o critério diagnóstico celular. O isolamento bacteriano foi positivo em 33.8% das amostras cultivadas no grupo PBE presumida.

## INTRODUCTION

Spontaneous bacterial peritonitis (SBP) is a bacterial infection arising in ascitic fluid when there is no evident intra-abdominal surgically treatable source of infection. The first description of SBP was in 1964.^1-3^ This common but severe complication in patients with liver disease can develop slowly and insidiously or remain clinically unrecognized until the appearance of symptoms like fever and abdominal pain. The mortality rate after a single episode ranges from 20 to 40%,^4,5^ and early diagnosis is required for adequate treatment and prevention of new episodes.

The incidence of spontaneous bacterial peritonitis in cirrhotic patients varies between 7% and 30% per year.^6,7^ The factors associated with higher risk are coexistent gastrointestinal bleeding, previous episodes of SBP and low levels of protein in ascitic fluid. Possible explanations for its pathogenesis include occurrences of bacterial overgrowth with deterioration of the intestinal barrier, lower intestinal motility, changes in local immune defense and lower activity of bacterial opsonization.^8-11^ Bacterial overgrowth precedes the key event in the pathogenesis of SBP: the bacterial translocation.^2,12-14^ This is defined as the passage of viable bacteria from the intestinal lumen to mesenteric lymph nodes and/or other extraintestinal sites across the intestinal-mucosal barrier.^8^ Non-enteric *Streptococcus sp* and Gram-negative aerobic enterobacteria like *Escherichia coli* (present in approximately 70% of cases) and *Klebsiella* sp are the microorganisms most commonly involved.^2,15-17^

Early detection of SBP is extremely valuable for patients, since the mortality rate among untreated patients is around 50%.^18^ The laboratory criterion most used for SBP diagnosis is an ascitic fluid neutrophil count ≥ 250 cells/mm^3^, in the absence of a source of intra-abdominal infection.^2^ Bacterascites (monomicrobial non-neutrocytic bacterascites) is the term used to describe the colonization of ascitic fluid by bacteria, with no evidence of local or systemic infection and no inflammatory reaction in the bacterial fluid (neutrophil count < 250/mm^3^ and positive bacterial culture). Culture-negative neutrocytic ascites is the term used to describe the clinical situation in which the ascitic fluid contains 250 or more neutrophils /mm^3^, but fluid cultures fail to grow any bacteria. This finding is considered to represent the expected 20% failure rate of cultures to isolate microorganisms.^2^ Despite the low complexity of laboratory tests used for diagnoses, prescriptions for antibiotic therapy are based on the most commonly involved pathogens and generally precede the bacterial culture results. Thus, an early diagnosis is highly desirable in order to avoid indiscriminate use of antibiotics, with potential induction of bacterial resistance or other complications relating to their use.

## OBJECTIVES

In this context, the aim of this study was to estimate the frequency of presumed cases of spontaneous bacterial peritonitis in the emergency rooms of a tertiary public university hospital, based on cytological criteria, and to assess the microbiological and biochemical profile of these peritoneal fluid samples.

## METHODS

### Subjects

We retrospectively analyzed laboratory data on 691 patients (431 males and 260 females; average age 58.1 years) from whom peritoneal fluid samples were collected in emergency rooms at a tertiary public hospital between November 2001 and November 2006. All the samples were received at the cytology laboratory containing a written diagnostic hypothesis of SBP on the laboratory test order. If more than one peritoneal fluid sample from any patient included was processed during the study period, only the first sample was taken into consideration in the study analysis, thus resulting in one sample for each patient.

The 691 samples were divided in two groups, based on their neutrophil count: group A (presumed spontaneous bacterial peritonitis: ≥ 250 neutrophils/mm^3^) and group B (no presumed spontaneous bacterial peritonitis: < 250 neutrophils/mm^3^). The study protocol was approved by the Institutional Ethics Committee.

### Methods

The following variables were evaluated: (1) patients’ sex and age; and (2) ascitic fluid characteristics such as: total number of nucleated cells and total and differential leukocyte count; presence of bacteria on Gram-stained slides and aerobic and anaerobic bacterial cultures; and total protein, albumin, adenosine deaminase (ADA), lactate dehydrogenase (LD) and glucose concentrations, when requested.

Ascites samples were collected by paracentesis using a sterile technique and the samples were immediately sent to the laboratory for analysis. For samples collected into EDTA (ethylenediaminetetraacetic acid) coated tubes,^19^ cells were counted manually in a Neubauer chamber^20^ and the cytological examination was performed on Leishman-stained smear slides. In cases with hemorrhagic fluid (red cells ≥ 10,000/ml), the neutrophil count was corrected by subtracting one neutrophil per 250 counted erythrocytes. For biochemical analysis, fluid samples collected into tubes containing gel separator plus clot activator were centrifuged and the supernatant was tested for total protein, albumin, globulin, lactate dehydrogenase and glucose concentrations, using a Roche modular analyzer (Roche Diagnostics, Roche, Somerville, United States).

ADA is an enzyme that is produced by lymphocytes and macrophages in response to T cell stimuli and is frequently increased in cases of peritoneal tuberculosis. The ADA level was measured using the Giusti modified manual method.^21^

Aerobic and anaerobic cultures were performed through bedside inoculation^19^ of fluid samples into Bactec culture bottles (BD Diagnostic Systems, Sparks, United States) and bacterial identification was performed by means of the Vitek automated identification system (BioMérieux Clinical Diagnostics, France).

### Statistical analysis

Differences between groups were evaluated using the Mann-Whitney test for non-categorical data and the chi-square test for categorical data, by means of the Statistical Package for the Social Sciences (SPSS) software (version 11.0, Chicago, United States). The data were presented as means ± standard deviations, unless otherwise indicated. Differences were considered significant if P < 0.05.

## RESULTS

Only 219 (31.7%) samples contained 250 or more neutrophils/mm^3^ (Group A, presumed SBP), while 472 samples (68.3%) had < 250 neutrophils/mm^3^ (Group B, no presumed SBP). We did not observe any statistically significant difference in relation to sex and age distribution between groups A and B. However, there was predominance of males in both groups (Table 1).

**Table 1.** Patients’ demographic data

	Group A Presumed SBP* (n = 219)	Group B No presumed SBP† (n = 472)	P
Age (mean ± SD)	58.3 ± 13.1	58.0 ± 14.5	0.911
Sex			
Males	139	292	0.749
Females	80	180	

* Ascitic fluid with ≥ 250 neutrophils/mm^3^;

† ascitic fluid with < 250 neutrophils/mm^3^;

SD = standard deviation; SBP = spontaneous bacterial peritonitis. Statistical tests: Mann-Whitney or chi-square; significant if P < 0.05.

Bacterioscopy, or Gram staining, is not obligatory in the routine workup for SBP, since its sensitivity is too low. Nevertheless, it was performed on 135 samples (61.6%) in group A and on 282 samples (59.7%) in group B, which yielded rates of positive findings of 12.6% and 1.1%, respectively (Table 2).

**Table 2.** Microbiological characteristics of ascitic fluids

	Group A Presumed SBP* (n = 219)	Group B No presumed SBP† (n = 472)	P
Bacterioscopy (Gram stain)			
Positive	17	3	< 0.001
Negative	118	279	0.226
Not performed	84	190	0.695
Bacterial culture			
Positive	63	24‡	< 0.001
Negative	123§	373	< 0.001
Not performed	33	75	0.870

* Ascitic fluid with ≥ 250 neutrophils/mm^3^;

† ascitic fluid with < 250 neutrophils/mm^3^;

‡ presumed bacterascites;

§ culture-negative neutrocytic ascites;

SBP = spontaneous bacterial peritonitis. Statistical tests: Mann-Whitney or chi-square; significant if P < 0.05.

Among the samples in group A, 33 (15%) were not subjected to bacterial culture. Among the cultured group A samples, 123 (66.2%) presented negative cultures and 63 (33.8%) had positive results (Table 2). The most prevalent agents were *Escherichia coli* (31.7%), followed by *Streptococcus pneumoniae* (7.9%), *Staphylococcus aureus* (7.9%), and *Klebsiella pneumoniae* (7.9%). The overall rate of positive findings of the genus *Streptococcus sp* was 23.8%. In group B, 75 samples (15.9%) were not subjected to bacterial culture. Among the cultured group B samples, 373 (94%) presented negative cultures and 24 (6%) had positive results that could be classified as potential bacterascites. The most prevalent agents in these cases were *E. coli* (20.8%), *S. epidermidis* (16.7%), *K. pneumoniae* (12.5%) and *Corynebacterium sp* (12.5%). A small number of cases displayed growth of atypical agents that are not usually associated with SBP, such as *Staphylococcus simulans*, *Staphylococcus hominis*, *Providencia stuartii*, *Citrobacter* *braakii* and *Streptococcus salivarus*.

Statistically significant differences were observed between groups A and B regarding the concentrations of glucose (109.4 ± 82.2 mg/dl versus 131.6 ± 76.4 mg/dl; P < 0.001) and LD (1466.8 ± 6169.5 U/l versus 255.2 ± 445.5 U/l; P < 0.001), and in relation to the percentages of total nucleated cells and some cell types.

The concentration of ADA, did not show any significant difference between the groups. On cytological examination, the number of nucleated cells was significantly higher in group A, mainly due to neutrophil predominance (Table 3).

**Table 3.** Biochemical and cytological characteristics of ascitic fluids

	Group A Presumed SBP* (n = 219)	Group B No presumed SBP† (n = 472)	P
Proteins (g/dl)	2.1 ± 1.7	1.8 ± 1.5	0.088
Glucose (mg/dl)	109.4 ± 82.2	131.6 ± 76.4	< 0.001
Albumin (g/dl)	1.0 ± 1.0	1.2 ± 6.3	0.147
LD (U/l)	1466.8 ± 6169.5	255.2 ± 445.5	< 0.001
ADA (UI/l)	30.1 ± 63.8	18.0 ± 29.3	0.065
Total nucleated cells/mm^3^	10082.5 ± 35181.3	300.6 ± 559.5	< 0.001
Leukocytes (%)	68.4 ± 25.1	43.3 ± 25.4	< 0.001
Macrophages (%)	28.1 ± 23.7	52.1 ± 25.9	< 0.001
Mesothelial cells (%)	3.5 ± 7.6	3.7 ± 7.0	0.909
Neutrophils (%)‡	79.8 ± 20.1	24.3 ± 25.4	< 0.001
Eosinophils (%)‡	0.6 ± 0.9	1.1 ± 3.9	0.420
Basophils (%)‡	0.0 ± 0.1	0.1 ± 0.2	0.100
Lymphocytes (%)‡	16.8 ± 19.0	69.5 ± 26.7	< 0.001
Monocytes (%)‡	2.6 ± 2.9	4.7 ± 6.1	< 0.001
Plasmocytes (%)‡	0.0 ± 0.0	0.0 ± 0.2	0.172

* Ascitic fluid with ≥ 250 neutrophils/mm^3^;

† ascitic fluid with < 250 neutrophils/mm^3^;

‡ leukocyte differential;

LD = lactate dehydrogenase; SBP: spontaneous bacterial peritonitis; ADA = adenosine deaminase. Statistical tests: Mann-Whitney or chi-square; significant if P < 0.05.

## DISCUSSION

In the present study, application of the cytological criterion for presumed SBP in clinically suspected patients resulted in positive findings in 219 (31.7%) of all the study samples. Another interesting finding was that 15.6% of the peritoneal fluid samples were not sent for bacterial culture, even though paracentesis had been performed due to clinically suspected infection. We found that 33.9% (63/186) of the cultured samples in group A presented positive cultures. This rate of positive cultures was lower than in the literature, in which rates ranging from 40 to 80% in confirmed SBP cases have been reported.^2,22^ In fact, cytological examination and bedside fluid inoculation into bacterial culture bottles are the two most accepted laboratory tests for investigation of SBP.^2,6^ For this reason, we emphasize the importance of ordering at least these two laboratory tests (cytology and cultures), in order to establish the diagnosis in suspected cases.

Regarding the microbial agents identified, our results were similar to those reported in literature,^2^ with *Escherichia coli* as the most prevalent agent.^17,23^ In a small number of cases with cellular criteria for SBP, we observed growth of some bacteria that are not usually related to this diagnosis. In such cases, it is important to rule out possible sample contamination during paracentesis or sample handling. False-positive results can lead to unnecessary antibiotic therapy, which could increase bacterial resistance to the antibiotics most used for treating SBP. In group B, only a small percentage of cases showed positive bacterial cultures, and most of them were for microbial agents that are not usually associated with SBP, which suggests that the peritonitis had non-spontaneous etiology.

We observed predominance of men over women among the study subjects, with an average age of around 60 years. This pattern was similar to what was observed in other reports,^24-26^ and probably reflects the classical natural history of patients with liver diseases who seek emergency care centers due to development of ascites. Most of these patients have cirrhosis with portal hypertension as a complication of a history of alcoholism or chronic hepatitis C virus infection, and both of these conditions are more prevalent among men.^25,26^

The finding of lower levels of glucose in the peritoneal fluid of patients with a presumed diagnosis of SBP probably reflects the consumption of this substance by bacteria, whereas the high concentration of LD reflects a high degree of peritoneal inflammation. An analogy can be made with parapneumonic pleural effusions, in which the high concentration of lactate dehydrogenase is one of the criteria used for classifying an effusion as complicated. In these cases, pleural fluid LD levels higher than 1,000 U/l, in association with decreased pH and glucose suggests clinical worsening and may be an indication for thoracic drainage.^27,28^ High LD activity (> 500 U/l) has been widely reported in cases of malignancy and tuberculous and pancreatic ascites but without enough sensitivity to distinguish it from liver disease. This makes low LD values unsuitable for ruling out malignancy, but indicates that elevated LD in fluid samples point towards causes other than liver disease.^29^ High levels of LD can also occur on SBP, as seen in the group A ascitic fluids, but can also occur in secondary bacterial peritonitis, which is frequently associated with intra-abdominal surgically treatable sources of infection,^30^ such as intestinal perforation. A study conducted by Boyer et al. found that ascitic fluids with two out of three of the characteristics of an exudate (LD > 400 U/l; fluid/serum LD ratio > 0.6; and fluid/serum total protein ratio > 0.5) tended to indicate a non-hepatic cause for the ascites.^31^ Since we did not review all the medical records, we could not identify possible cases of secondary peritoneal infection.

The management of ascitic patients is to a great extent influenced by laboratory test results. In clinical practice, since the collection of peritoneal fluid samples can be a time-consuming and cumbersome procedure, the use of this biological material needs to be optimized by ordering relevant tests and paying special attention to pre-analytical best-practice procedures in order to increase the reliability of test results. Some of these recommended procedures are: (1) bedside inoculation of ascitic fluid into culture bottles and referral to a quality-certified microbiology laboratory; (2) for adequate cell counting analysis, collection of ascitic fluid into EDTA-coated tubes, in order to avoid fibrin formation and cell clumping, and (3) immediate transportation of samples to the laboratory, in order to avoid time and temperature-related pre-analytical errors, especially in biochemical tests. In our laboratory, we routinely perform body cavity fluid cell counting in Neubauer chambers (manual technique), instead of using automated counting devices. The latter could be an alternative,^19^ but these devices show poorer accuracy, particularly for fluid samples with low cell counts.^20^

Among the limitations of our study, it should be noted that we did not review the patients’ clinical records to check for any underlying clinical conditions such as recent gastrointestinal bleeding or abdominal surgery, or to investigate any secondary sources of peritoneal infection, cirrhosis and other causes of ascites. Furthermore, since we did not check for concomitant or recent use of antibiotics, we were unable to estimate the impact of antibiotic use on negative results from cultures. However, because the samples were sent to the laboratory as probable SBP cases, we supposed they represented a heterogeneous group of patients, mostly with cirrhosis, which is the major underlying condition that raises the suspicion of SBP in patients with ascites.

In any event, it is worth emphasizing to clinicians the importance of proper sample collection and management, as well as correct ordering of relevant laboratory tests for investigating suspected SBP cases, not only to achieve early diagnosis, but also to avoid unnecessary antibiotic administration.

## CONCLUSIONS

In conclusion, although SBP is a commonly encountered disease in emergency medical rooms, the number of presumed cases in a tertiary university hospital in Brazil was 31.7% of all cases of suspected peritoneal fluids analyzed over a five-year period.
